# Field of daydreams? Integrating mind wandering in the study of sluggish cognitive tempo and ADHD

**DOI:** 10.1111/jcv2.12002

**Published:** 2021-03-20

**Authors:** Stephen P. Becker, Russell A. Barkley

**Affiliations:** ^1^ Cincinnati Children's Hospital Medical Center Division of Behavioral Medicine and Clinical Psychology Cincinnati Ohio USA; ^2^ Department of Pediatrics University of Cincinnati College of Medicine Cincinnati Ohio USA; ^3^ Department of Psychiatry Virginia Commonwealth University Medical Center Richmond Virginia USA

## Abstract

In this editorial perspective, we consider the potential conceptual and empirical overlap between the research on mind wandering, particularly in its pathological extreme, and that on sluggish cognitive tempo (SCT) as it has diverged from research on attention‐deficit/hyperactivity disorder. The more advanced state of research findings on the nature and correlates of mind wandering relative to that of SCT is used to suggest a variety of avenues of investigation into SCT, such as its phenomenology, positive and negative correlates, research methods, theory building, and potential to inform interventions. These and other avenues drawn from the field of mind wandering are likely to prove fruitful in further revealing the nature of SCT and its relationship to mind wandering.

One of Tim's dominant characteristics is that he is extremely spacey most of the time… He tends to be hypoactive, passive, and unengaged and if not actively engaged by someone will fade out and daydream… When we read the “checklist” that has been created for SCT it was as though Tim was being described for the first time on paper.E‐mail from a concerned mother

In recent decades, two literatures have advanced in parallel that seem enticingly overlapping. In cognitive psychology and neuroscience, there has been substantial advancement in understanding the nature of mind wandering, largely driven by interest in understanding the brain's default mode network (Christoff et al., 2016). In clinical psychology and psychiatry, there has been growing interest in sluggish cognitive tempo (SCT), another apparent attention deficit, given its relevance for and contrast with attention‐deficit/hyperactivity disorder (ADHD) and other mental disorders (Becker et al., 2016). Although they arose independently, the fields share conceptual and empirical overlap. First noted a decade ago (see Becker & Barkley, 2018), this overlap was implied in recent empirical findings on SCT. Here, we describe how mind wandering and SCT are conceptually related and offer a research agenda to integrate these two fields to advance theory and practice.

## CONCEPTUAL AND EMPIRICAL OVERLAP IN SCT AND MIND WANDERING

Mind wandering is defined as a type of spontaneous thought lacking strong constraints on the thought contents that is distinct from but similar to daydreaming (Christoff et al., 2016), with a daydreaming frequency scale often used to measure mind wandering and some scholars referring to them as synonymous. Likewise, although SCT includes mental confusion, slowed behavior, and sleepiness, “daydreams” is used most frequently as a cardinal item of SCT (Becker et al., 2016).^1^ Both conditions appear to involve a decoupling of attention from the external environment and its redirection to various forms of mental content. Much of the research on SCT has sought to distinguish it from ADHD inattentive symptoms and their clinical correlates. It has been suggested that ADHD inattention may represent an attentional problem characterized by external distractibility whereas SCT may represent one characterized by internal (mental) distractibility. Here again, this parallel has striking resemblance to conceptualizations of mind wandering (Smallwood & Schooler, 2015). Although it would be overly simplistic to suggest that SCT and ADHD can be cleanly carved into internal versus external distractions, this heuristic may prove to be a useful starting point to advance discovery on the distinction and covariation of SCT and ADHD.

Until recently, there were no data directly linking mind wandering and SCT. There are now two studies demonstrating SCT to be associated with greater mind wandering (Fredrick & Becker, 2020; Fredrick et al., 2020). These studies found the association between ADHD inattentive symptoms and mind wandering to be largely eliminated when SCT symptoms were included in the model. Notably, both studies used a cross‐sectional design and assessed mind wandering using a self‐report measure of daydreaming frequency often used in studies of mind wandering. Nevertheless, these initial findings call into question whether the link between mind wandering and ADHD is as robust as previously believed (Bozhilova et al., 2018).

### Parallel findings linking SCT and mind wandering with functional outcomes

Beyond conceptual links, SCT and mind wandering are also associated with similar domains of functioning and maladjustment. Perhaps most consistently, they are both associated with increased negative mood symptoms, including depression (Becker & Barkley, 2018; Smallwood & Schooler, 2015). Although unstudied in the SCT field, the content and temporal nature (e.g., thinking about the past or future) of mind wandering experiences appear to be highly relevant in establishing whether mind wandering predicts negative mood and depressive symptoms (Smallwood & Schooler, 2015).

Mind wandering also negatively impacts reading comprehension, likely because the latter requires ongoing monitoring and encoding of inputs (Smallwood & Schooler, 2015) and actively holding what is read and understood in mind (working memory). Mind wandering may cause a decoupling of attention from such external monitoring of text as well as competing for working memory capacity. A recent study found SCT symptoms to prospectively predict poorer reading (including reading comprehension) whereas ADHD inattention uniquely predicted poorer math achievement (Becker et al., 2018).

SCT is reliably associated with social difficulties, and social withdrawal and isolation in particular (Becker & Barkley, 2018). It may be that individuals with elevated SCT symptoms find complex social situations to be stressful, aversive, or even overwhelming, and withdraw or escape into daydreams as a result. Drawing from the mind wandering literature, it may be important to evaluate the nuance of who or what individuals with SCT daydream about, as daydreaming about people not close to us is associated with greater loneliness and lower perceived social support whereas daydreaming about people close to us is not (Mar et al., 2012).

### Mind wandering as a framework for advancing the study of SCT

Here are five ways in which investigators interested in SCT can draw from the mind wandering literature to rapidly advance the field:

1. **Embracing phenomenology**. The field would greatly benefit from investigating the content and context of daydreaming and mind wandering behaviors among individuals with elevated SCT symptoms. What are individuals with clinical elevations in SCT, including frequent daydreaming, thinking about? We do not know. What we do know from the mind wandering literature is that content, context, and temporal orientation matter for understanding the nature of mind wandering and possible associations with functional outcomes (Smallwood & Schooler, 2015). Mind wandering episodes are more frequently temporally oriented toward the future than to the past, with a retrospective bias in mind wandering associated with lower mood (Smallwood & Schooler, 2015). Are individuals with SCT more prone to ruminating about the past? In addition, to what degree are they aware that their minds have wandered (meta‐awareness), as tuning out (mind wandering with awareness) is related to poorer task performance and depressive symptoms than zoning out (mind wandering without awareness)? Understanding the temporal orientation, context, and meta‐awareness of daydreaming content may allow for greater specificity in determining under what conditions SCT symptoms are associated with emotional, social, and/or academic outcomes.
2. **Balancing negative and positive outcomes**. Much of the investigation of mind wandering and SCT has focused on their associations with negative outcomes, including negative affect, loneliness, and academic and occupational difficulties. It is critical to understand the burden of SCT, as any impact of SCT on functional impairment is typically what matters most to parents, teachers, and individuals themselves. Yet there is increasing interest in the potential benefits of mind wandering, focusing largely on creativity, future planning, social endeavors, and meaning‐making (Smallwood & Schooler, 2015). We are not aware of any studies that have tested SCT in relation to these positive attributes, though parents of children with elevated SCT often comment on the potentially positive attributes of daydreaming. As noted previously, “In considering SCT, although daydreaming itself is not pathological and is beneficial for play, imagination, and creativity, the duration, intensity, and content of daydreams may be especially important for clinical assessment and discrimination” (Burns et al., 2020, p. 467). Grounded in a developmental psychopathology framework, there is a need for empirical research examining in tandem the costs and benefits of SCT.
3. **Bridging methods**. To expand beyond the use of subjective rating scales and to promote multi‐method investigations, the study of SCT would greatly benefit from incorporating methods that have become standard in the mind wandering field. Mind wandering is most frequently studied using experience sampling methods, often using the sustained attention to response task (SART). While completing the SART, a participant may be asked to indicate when they notice that their thoughts have wandered from the task (self‐caught method) or be periodically asked as to their mental state (probe‐caught method). They may be queried instead at the end of the SART so as to not interfere with the time course of the task (retrospective method). The participant may further be asked to indicate whether a mind wandering episode was spontaneous or deliberate, or the content of their mind wandering (e.g., about the past or future). Additional experimental manipulations may also be used, for example by administering the SART under high or low working memory conditions, under positive or negative mood inductions, or with difficult (standard SART with randomly presented numbers) or easy (numbers presented in order) versions of the task. Response sampling via personal smart technologies (phones, tablets, watches, etc.) has been used to explore the frequency and nature of mind wandering in natural ecologies. And clinically, patients with pathological mind wandering have been queried concerning its contents and the results categorized as to themes which are then associated with other clinical correlates. Moreover, the decreased monitoring of the external environment during periods of excessive mind wandering is associated with poorer episodic recall of events and their details (Smallwood & Schooler, 2015). Is this true for SCT? Certainly, our clinical experience concerning the reports of others about those having SCT suggests this is likely to be the case. Finally, mind wandering has been shown to be more likely to occur when tasks that must be performed are overlearned and routine, thus demanding little goal directed cognitive control and working memory (executive functioning) while being less likely during the performance of novel tasks. To our knowledge this association of SCT symptoms with various task conditions has not yet been explored but would be fruitful to do so to further evaluate whether, and when, SCT symptoms are associated with poorer neurocognitive functioning. Using these and other methods has the potential to advance the field's understanding of SCT symptoms by incorporating experimental and behavioral/ecological findings with existing observational findings.
4. **Leveraging units of analysis to build theory**. Beyond the commonly used SART, mind wandering is associated with a host of objective measures of behavioral and brain functions, including response time variability, eye movements and pupil dilation, electroencephalogram patterns (including reduced P3 amplitude), and blood‐oxygen‐level dependent signal recording during functional magnetic resonance imaging (fMRI). Of these, fMRI has been most frequently examined, with investigations converging in demonstrating mind wandering to be associated with activation of the default mode network (DMN), including the medial prefrontal cortex, posterior cingulate cortex, medial temporal lobe, and bilateral inferior parietal lobe, as well as non‐DMN regions such as the frontoparietal control network (Christoff et al., 2016). These findings provide fertile ground for examining the potential brain basis and behavioral correlates of SCT, both independently and in relation to ADHD. Integrating multiple units of analysis, including brain circuitry, physiology, and behavioral tasks, alongside existing self/informant‐report measures of SCT, will be important for situating findings within broader literatures and, perhaps most importantly, essential for building comprehensive theories of SCT.
5. **Informing intervention**. Meditation involves practice to train one's ability to maintain focus or attention on a particular object or thought, and therefore is a natural starting place for interventions aiming to reduce mind wandering (Smallwood & Schooler, 2015). Mindfulness‐based approaches, within a larger cognitive‐behavioral framework, may also be a fruitful avenue for intervention for youth and adults with SCT (Becker & Barkley, 2018). It will be especially important for intervention trials to not only evaluate whether mindfulness‐based interventions reduce SCT symptoms, but also improve both behavioral task performance (e.g., SART performance) and reduce functional impairments (e.g., social or academic impairment). As for pharmacological intervention, clinical reports suggest that serotonergic reuptake inhibitors having some use in managing ruminative thoughts and obsessive‐compulsive disorder (fluvoxamine) may be helpful for managing pathological mind wandering; one of several potential drug treatments yet to be explored in the field of SCT.

## CONCLUSION

This paper outlines some of the numerous ways that the study of SCT and, by default, ADHD will be advanced by a careful consideration of mind wandering, its nature, correlates, research methods, and interventions, as they may be applicable to our understanding of SCT. We propose that it is well past time for mind wandering and SCT to cease operating in parallel research silos, and instead, to integrate these fields in an effort to better understand the nature, impacts, and intervention strategies for individuals who experience excessive daydreaming.

## CONFLICT OF INTEREST

During the previous 12 months, Dr. Becker declares royalties for a book from Guilford Press. He is also a Joint Editor for JCPP *Advances*. During the previous 12 months, Dr. Barkley declares royalties for books, a newsletter, and rating scales from Guilford Press and the American Psychological Association, and royalties for Internet CE courses from Premier Educational Seminars, Inc., and ContinuingEdCourses.net. He also has received a speaker fee from Medice Pharmaceutical Co. (Germany). [Corrections made on 22 June 2022, after first online publication: This Conflict of Interest statement has been updated in this version.]

## AUTHOR CONTRIBUTIONS

Both authors contributed to the conceptualization, writing, and editing of this editorial perspective.

## Data Availability

This editorial perspective does not report on any new data.

## ENDNOTES

^1^
It is important to keep in mind that in this editorial we focus on the daydreaming aspect of sluggish cognitive tempo, which although central to the construct is not all‐encompassing and does not include other cognitive (e.g., mental confusion) and behavioral (e.g., sleepy, sluggish) symptoms.
